# Drug Eruptions: Their Nature and Varieties—II

**Published:** 1911-01-14

**Authors:** J. H. Stowers

**Affiliations:** Vice-President of the Section of Dermatology, Royal Society of Medicine; late Physician to the Department for Diseases of the Skin, N.W. London Hospital; formerly President of the Dermatological Society of Great Britain and Ireland.


### Hospital Clinics.

### DRUG ERUPTIONS: THEIR NATURE AND VARIETIES—II.

By J. H. STOWERS, M.D., Vice-President of the Section of Dermatology, Royal Society of Medicine; late Physician to the Department for Diseases of the Skin, N.W. London Hospital; formerly President of the Dermatological Society of Great Britain and Ireland.

(Lecture at the Polyclinic, Chenies Street, W.C.)

Concerning the general characters of drug eruptions taken collectively, it is remarkable that all the lesions of the skin with which we are familiar may separately or together be produced, their development being gradual or rapid according to the many varying conditions which exist, such as quality and quantity of the drug, mode of application, idiosyncrasy (general or local), conditions of health (with or without impaired function of excretory organs), climate, occupation, and the like.

Polymorphism constitutes a distinctive feature of drug eruptions, as Morrow has pointed out. The erythematous varieties are in point of time the first to appear, and later those which are papular or tubercular in form, constituting nodules or bosses of very considerable size, and possibly followed by ulceration and sloughing. By "tubercular" is meant an hypertrophied papule or solid elevation of the skin wholly apart from tuberculosis. Some cases of bromism illustrate these developments to a very marked degree. The intensity and extent of the eruption are not always commensurate with the quantity administered, some persons presenting maximum effects from minimum doses. Sufficient similarity of effects or uniformity are, however, met with to allow of rough groupings. The results of such drugs as chrysarobin, croton oil, and tar are sufficiently typical to be recognised at once in the majority of instances. Again, it is well known that eruptions simulating scarlet fever and measles are invariably produced by such drugs as belladonna and antipyrin respectively (Morrow). One of the most remarkable instances of a morbilliform eruption due to a drug I have seen was caused by chloral hydrate, but this is not constant.

### Veronal Rash.

About two years ago I was asked to see a lady thirty-five years of age at West Hampstead with her medical attendant, in consequence of an illness attributed to scarlet fever. She lived with her husband and only daughter in a flat, and as in those circumstances an infectious ailment was of serious importance, the question was raised as to the necessity and propriety of her removal. All steps had been taken as to the control of possible infection, and the girl had already been sent from home. The patient had some limited constitutional disturbance, and being neurotic was in a state of marked mental anxiety, with curious variations of body temperature. The doctor regarded the case as of unusual character, but had not been informed that the patient was the subject of insomnia and had taken remedies on her own account. By careful questioning as to drugs we discovered together that she had been recommended veronal, and had taken gr. x. tablets of this for many consecutive nights. The scarlatiniform eruption from which she suffered was entirely due to this drug. Itching was a prominent symptom.

### Varieties of Drug Rashes.

The late Dr. Radcliff Crocker, whose death we so deeply deplore, enumerated the various forms of drug eruptions under the following thirteen heads, namely: erythemata, vesicular, bullous, urticarial, pustular, purpuric, gangrene, abscess, furuncles, persistent desquamation, ecthyma, zoster, and pigmentation, and added three others, or those which have appearances more or less simulating: eczema, psoriasis, and pityriasis rubra.

It must be remembered, however, that the same drug is capable of producing at different stages of its influence very dissimilar appearances. Quinine is followed by erythema, vesicles, and bullæ, eruptions of urticarial and purpuric kind, exfoliation, abscess, and even gangrene.

I have seen in the person of a woman who had large doses of quinine for a considerable period for the treatment of malarial cachexia several sloughs on the trunk and limbs which might easily have been mistaken for syphilitic ulceration.

The more sensitive surfaces, such as the face, flexures of joints, etc., are naturally more prone to the effects of local irritants than other parts, besides which, as has been pointed out, the eruption may occur quite distant from the seat of contact. The course of the eruption, which is generally variable, may cease to develop after a certain stage, in spite of the persistent administration of the drug. This I have seen in cases of bromism.[1] Sometimes, however, the cutaneous manifestation continues for a considerable period after the ingestion of the drug has ceased. Occasionally a seeming predilection for cicatricial tissue has been noted in cases of eruption due to the bromides, including vaccination scars in young children.

The majority of drug eruptions on general ætiological and other grounds must be included in the list of toxic erythemata.

Formerly but a limited number of medicinal agents were recognised as causing cutaneous lesions (namely, roseola and urticaria after the ingestion of a few drugs) of which copaiba was probably the most common, but of late years the list of drugs capable of producing eruptions of the skin through vasomotor nerve influence has been largely added to, particularly in those prone to eczematous manifestations, whose cutaneous proclivities may be transmitted through successive generations, and in persons of so-called scrofulous habit and lymphatic temperament. That a textural predisposition often exists is undeniable. A clinical distinction, however, should be drawn even in eruptions from the external application of drugs, writes Morrow, "between those which are constant and regular and those which are variable and irregular, the former depending mostly upon the drug itself, the latter upon the predisposition of cutaneous tissues."

Iodide Eruptions.

The eruptions following the use of iodides may be mentioned as being strangely variable, sometimes acneiform only, sometimes bullous and pustular, sometimes tubercular, and followed by the formation of subcutaneous nodules. In a remarkable case I saw with the late Dr. Sangster, so unusual were the manifestations that it was impossible by appearance alone to distinguish them from dermato-syphilis. However, with the discontinuance of the drug the lesions disappeared. In this relation I recall a case in my own practice which is worth remembering, not merely on account of the importance of accurate diagnosis, but as showing what additional risks may be inflicted upon a patient when uncertainty prevails.

A gentleman from the North of England consulted me for a dermato-syphilide. He had had previous treatment, including iodide of potassium, but the doses were small, and, as he anticipated matrimony within twelve months of the date of my seeing him, he was specially anxious for a rapid cure.

The suggestion I made was that he should increase the dose, for a period, to 10 grains three times a day, and if matters did not progress satisfactorily I would reconsider his case. He left London immediately afterwards. Several weeks later I casually met his brother, an old patient of mine, through whose influence, I believe, he originally came to me. He told me that the patient returned to town a few days previously feeling very unwell, and having a pustular eruption all over his body. He feared it was smallpox, so a doctor was sent for at once and he confirmed the opinion. As my friend's house was at Clapham, an ambulance was sent for and the patient was immediately removed to the Stockwell Fever Hospital.

On suggesting the possibility of an iodide rash, I was allowed to visit him. The resident medical officer at the hospital frankly confessed some doubt about the case, but he felt it incumbent upon him to admit the patient, which he did. A closer investigation satisfied us that he was merely the subject of an acneiform pustular eruption due to iodide of potassium, and he obtained his discharge forthwith.

Instead of keeping to the prescribed dose the patient had *trebled* it upon his own responsibility, thus taking 90 grains per diem instead of 30 grains. As an aid to diagnosis, *persistence* and *recurrence* must always be borne in mind. Besides the influence of "local idiosyncrasy" it has been contended that part of the disturbing action of certain agents expends itself upon the skin, and also that some eruptions result from irritation of certain structures—namely, the sebaceous glands through which a particular drug is eliminated in part, while others are due to changes in the blood, including disorganisation of blood corpuscles.

It is also well known that both functional and structural disorders of the kidneys predispose to the production of drug eruptions by means of defective elimination. The late Dr. Parkes, of Netley Military Hospital, and later Sir William Roberts and others, published years ago special observations in this direction. Numerous writers have been led to the conclusion that the sebaceous glands are the seat of primary structural change in eruptions due to bromism and iodism, but instances have been recorded showing that this is not constant. Dr. Thin[2] described the sections he made of the skin of a patient who died during the time that an extensive eruption, due to iodide of potassium, was present, and he reported that the "sebaceous glands were unaffected, the main pathological condition being the blocking of vessels at certain points, leading to rupture, and the formation of varying degrees of the local lesions." Drs. Arloing[3] and Alfred Salter[4] have published some remarkable observations on the subject of bacterial toxin elimination by means of the sweat, in health and disease, which are worthy of careful study.

Lastly, some drugs act in a specific manner upon the nervous system, and localised neuritis may even result from their toxic effects, arsenical herpes probably being the commonest example. Many observers have written in detail upon this subject.

*(To be continued.)*

[1] *Vide Clinical Society's Transactions*, vol. xviii., with plate, including paper by the late Dr. Carrington; also *Transactions Pathological Society*, vol. xiii., with plate, and paper by Dr. Lees.

[2] *Royal Medical Chirurgical Society's Transactions*, November 1898.

[3] *Lancet*, September 25, 1897.

[4] *Lancet*, September 4, 1897.

